# The Marine Fungal Metabolite, Dicitrinone B, Induces A375 Cell Apoptosis through the ROS-Related Caspase Pathway

**DOI:** 10.3390/md12041939

**Published:** 2014-04-02

**Authors:** Li Chen, Mei-Wei Gong, Zhen-Fei Peng, Tong Zhou, Min-Gang Ying, Qiu-Hong Zheng, Qin-Ying Liu, Qi-Qing Zhang

**Affiliations:** 1 Institute of Biomedical and Pharmaceutical Technology, College of Chemistry and Chemical Engineering, Fuzhou University, Fuzhou 350002, China; E-Mails: ibptcl@fzu.edu.cn (L.C.); 742946642@qq.com (M.-W.G.); zhenfei2001@163.com (Z.-F.P.); vickychou90220@163.com (T.Z.); zhangqq@126.com (Q.-Q.Z.); 2 Fujian Provincial Key Laboratory of Tumor Biotherapy, Fujian Provincial Tumor Hospital, Fuzhou 350014, China; E-Mail: yingmg@163.com (M.-G.Y.); 3 Institute of Biomedical Engineering, Chinese Academy of Medical Science, Peking Union Medical College, Tianjin 300192, China

**Keywords:** dicitrinone B, marine-derived fungus, human malignant melanoma cell A375, anticancer activity, apoptosis

## Abstract

Dicitrinone B, a rare carbon-bridged citrinin dimer, was isolated from the marine-derived fungus, *Penicillium citrinum*. It was reported to have antitumor effects on tumor cells previously; however, the details of the mechanism remain unclear. In this study, we found that dicitrinone B inhibited the proliferation of multiple tumor types. Among them, the human malignant melanoma cell, A375, was confirmed to be the most sensitive. Morphologic evaluation, cell cycle arrest and apoptosis rate analysis results showed that dicitrinone B significantly induced A375 cell apoptosis. Subsequent observation of reactive oxygen species (ROS) accumulation and mitochondrial membrane potential (MMP) reduction revealed that the apoptosis induced by dicitrinone B may be triggered by over-producing ROS. Further studies indicated that the apoptosis was associated with both intrinsic and extrinsic apoptosis pathways under the regulation of Bcl-2 family proteins. Caspase-9, caspase-8 and caspase-3 were activated during the process, leading to PARP cleavage. The pan-caspase inhibitor, Z-VAD-FMK, could reverse dicitrinone B-induced apoptosis, suggesting that it is a caspase-dependent pathway. Our data for the first time showed that dicitrinone B inhibits the proliferation of tumor cells by inducing cell apoptosis. Moreover, compared with the first-line chemotherapy drug, 5-fluorouracil (5-Fu), dicitrinone B showed much more potent anticancer efficacy, suggesting that it might serve as a potential antitumor agent.

## 1. Introduction

Malignancies are one of the most serious diseases that damage human health in the modern world. Although the efficacy of chemotherapy for the majority of cancer types has improved over the last three decades, the high toxic effects of chemotherapeutic drugs, causing a severe reduction in quality of life, are still formidable problems in clinical medicine [1]. Therefore, many researchers have begun to investigate natural products, due to their antitumor activities and low side-effects [2].

Natural products from microbes have been a remarkable source of antitumor agents [3], in view of their advantages, such as fast growth and easy culture and purification. Since the discovery of antinomycin around 1940, many microbial products have been approved as antitumor drugs, including actinomycin d, anthracyclines, bleomycin, mitomycin c, anthracenones, enediynes and epothilones. New techniques, like the utilization of uncultivated microorganisms and metagenomics, have exhibited great promise for discovering new and powerful antitumor drugs [3]. 

Citrinin dimers are naturally occurring polyketone compounds from microbes, which have been reported to possess many kinds of bioactivities, such as enzyme inhibitory [4], antifouling [4], antioxidant [5], antitumor [6,7,8,9] and antimicrobial [9] properties. Among these bioactivities, the antitumor property is getting the most attention. However, the detailed molecular mechanisms are still not fully understood, because of the trace amounts of natural products and the difficult total synthesis. In an effort to search for antitumor compounds from marine-derived fungus, a rare carbon-bridged citrinin dimer, dicitrinone B, was purified from a lot of secondary metabolites. It was first reported by Du *et al.* in 2010 [6] to show moderate cytotoxic activities against four tumor cell lines, including leukemia (HL-60 and MOLT-4), liver (BEL-7402) and lung (A-549) tumor cells. Due to the trace amounts of dicitrinone B, they only evaluated cytotoxicity and cycle arrest on HL-60 cells. No information about the chemo-sensitization effects is available. With enough natural products available, we evaluated the inhibition ability of dicitrinone B on different kinds of human tumor cell lines and investigated the detailed molecular mechanisms of the antitumor activity of dicitrinone B.

As we know, most of the cytotoxic anticancer drugs in current use could induce apoptosis in susceptible cells [10]. Apoptosis is an important physiological process responsible for maintaining the balance of homeostasis. A defect in apoptosis plays a pivotal role in aberrant cell survival and tumorigenesis [11]. Cancer cells evade apoptosis by downregulation of death receptors, overexpression of anti-apoptotic proteins or reduced expression of pro-apoptotic proteins and caspases [12]. Induction of apoptosis is currently recognized as an active strategy to arrest the proliferation of cancer cells [13,14]. Our data suggest that dicitrinone B triggers apoptosis through the reactive oxygen species (ROS)-related caspase pathway, which is regulated by Bcl-2 family proteins in an A375 human malignant melanoma cell model.

## 2. Results and Discussion

### 2.1. Structural Elucidation of Dicitrinone B

**Figure 1:**
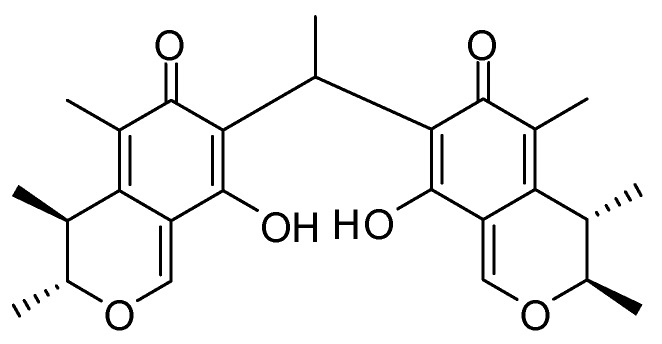
Chemical structure of dicitrinone B.

The purified compound, the purity of which was >95%, based on the peak area of all components absorbed at each specific wavelength in HPLC analysis (Supplementary Figure S1), was confirmed by comparing its HRESIMS (Supplementary Figure S2), ^1^H and ^13^C NMR data to the literature report (Supplementary Figures S3 and S4) [6]. The compound was identified as dicitrinone B, illustrated in Figure 1.

### 2.2. Dicitrinone B Inhibits the Proliferation of Multiple Tumor Types

**Figure 2:**
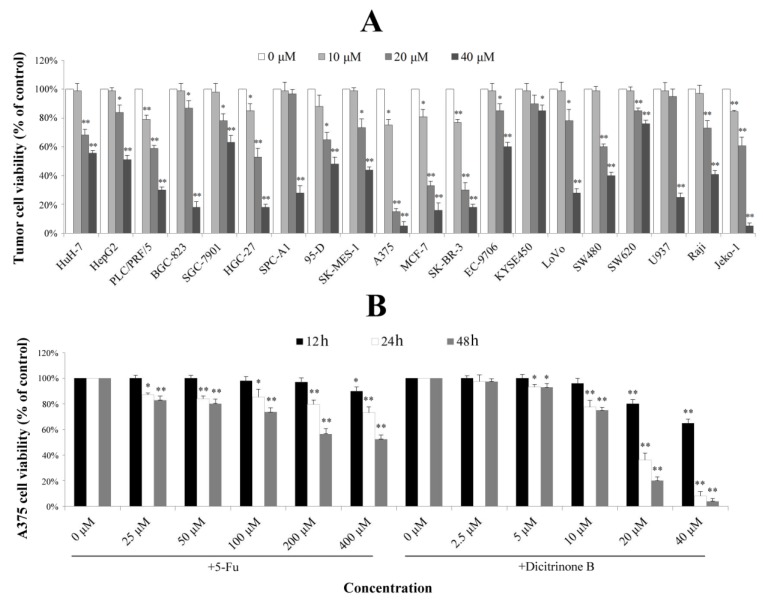
Dicitrinone B inhibits the proliferation of multiple tumor types. (**A**) The effects of dicitrinone B on multiple tumor types by WST-1 assay after tumor cells exposure to zero, 10, 20 and 40 μM of dicitrinone B for 48 h. (**B**) A375 cells were treated with dicitrinone B (zero, 2.5, five, 10, 20 and 40 μM) or 5-fluorouracil (5-Fu) (zero, 25, 50, 100, 200 and 400 μM) for 12, 24 and 48 h. The viability of cells was determined by WST-1 assay. The data are presented as the means ± SD from at least five independent experiments. Significant differences between the treatment and control groups are indicated as * (*p* < 0.05) or ** (*p* < 0.01).

A previous study reported that dicitrinone B showed moderate cytotoxic activities against four tumor cell lines [6]. To examine its effect on other tumor cells, twenty tumor cell lines derived from eight different types of tumors were used for evaluating cell growth inhibition. As shown in Figure 2A, different tumor cell lines had different levels of proliferation inhibition after being treated with gradient concentrations of dicitrinone B for 48 h. The IC_50_ values of the three most sensitive cell lines, including malignant melanoma cell line A375 and breast cancer cell lines SK-BR-3 and MCF-7, were 13.38 μM, 14.28 μM and 15.70 μM, respectively. Since the malignant melanoma cell line, A375, was the most sensitive, we finally chose it as the target cell line for further study. The effect of the first-line chemotherapy drug, 5-fluorouracil (5-Fu), was also tested as a positive control on A375 cells. The results showed that the viability of A375 cells by dicitrinone B presented a slightly lower change compared to the cells treated with ten times the concentration of 5-Fu after 12 h of treatment; when the exposure time reached 24 h, the viability of cells treated with 20 μM and 40 μM of dicitrinone B was more significantly decreased compared to that of cells treated with ten times the concentration of 5-Fu, and it dropped to 36.17% and 8.16%, respectively; the viability for 48 h under dicitrinone B treatment presented slightly lower growth than did the 24-h group and still presented a stronger effect compared to 5-Fu (Figure 2B). The IC_50_ of dicitrinone B for 24 h was 16.61 μM, while the IC_50_ of 5-Fu was more than 40 μM, revealing that dicitrinone B treatment inhibits A375 cell growth in a dose-and time-dependent manner and has more potent anticancer activity than 5-Fu.

**Figure 3:**
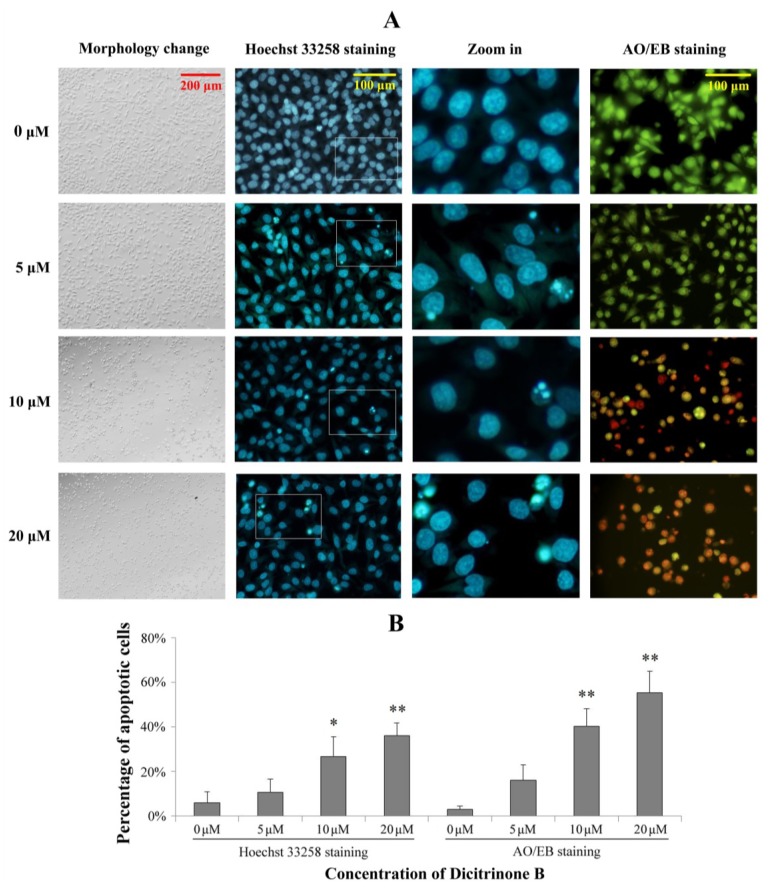
Dicitrinone B induces significant apoptotic morphological changes. (**A**) After being exposed to zero, five, 10 and 20 μM of dicitrinone B for 24 h, A375 cells were stained by Hoechst 33258 or acridine orange/ethidium bromide (AO/EB). (**B**) Quantification of the proportion of apoptotic cells detected by Hoechst 33258 staining and AO/EB staining. Data are presented as the mean ± SD from three independent experiments. Significant differences between the treatment and control groups are indicated as * (*p* < 0.05) or ** (*p* < 0.01).

### 2.3. Dicitrinone B Induces Significant Apoptotic Morphological Changes in A375 Cells

To determine whether the growth inhibitory activity of dicitrinone B was related to the induction of apoptosis, a morphological assay was performed using the Hoechst 33258 staining and acridine orange/ethidium bromide (AO/EB) staining. As shown in Figure 3, the proportion of apoptotic cells with chromatin condensation and apoptotic bodies were increased to 26.71% and 35.92% after exposed to 10 and 20 μM of dicitrinone B, while the control group was mainly living cells with normal nuclei. The AO/EB double staining results also showed green early apoptotic cells, with nuclear margination and chromatin condensation occurring when treated with 5 μM of dicitrinone B, and 40.24% and 55.15% of orange later apoptotic cells with fragmented chromatin were observed when the concentration of dicitrinone B raised to 10 μM and 20 μM, respectively, indicating that dicitrinone B could cause obvious cellular morphological change, such as cellular shrinkage, and induce apoptosis in A375 cells.

**Figure 4:**
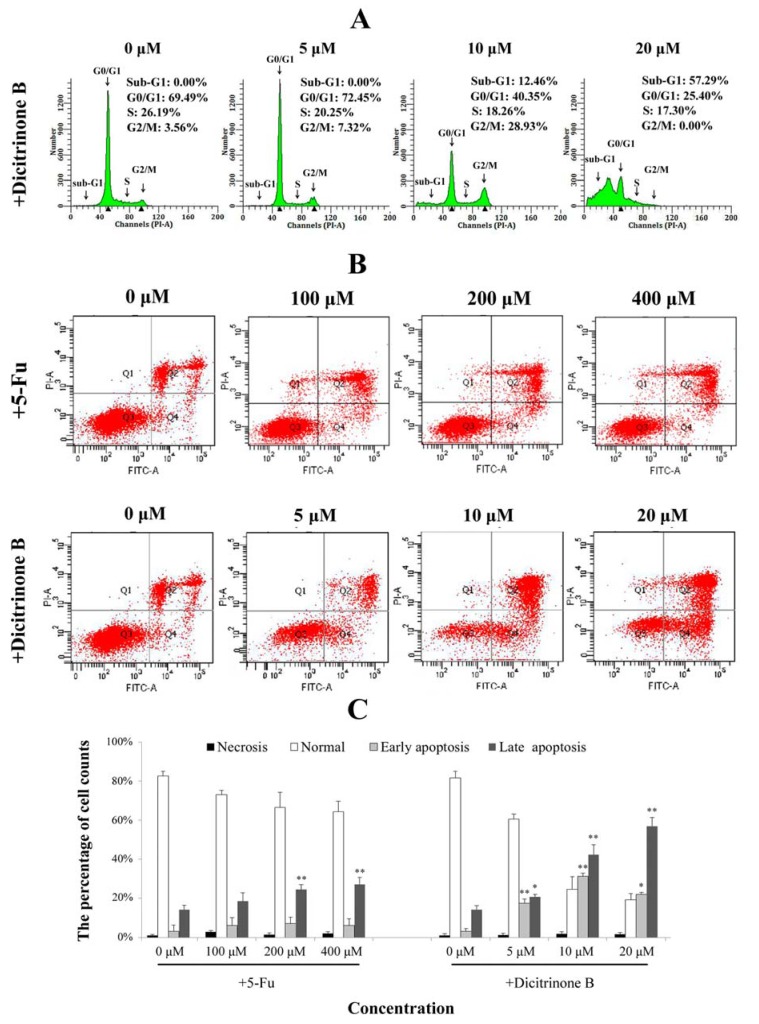
Dicitrinone B induces A375 cell apoptosis. (**A**) Cell cycle analysis by flow cytometry. After being treated with dicitrinone B (zero, five, 10 and 20 μM) for 24 h, cells were fixed in ethanol and stained with propidium iodide. DNA content was determined. (**B**) A375 cells were treated with dicitrinone B (zero, five, 10 and 20 μM) for 24 h and were analyzed with Annexin-V/PI staining by flow cytometry, with 5-Fu (zero, 100, 200 and 400 μM) as a positive control. Q1 represents necrotic cells; Q2 represents non-viable apoptotic cells; Q3 represents normal cells; and Q4 represents viable apoptotic cell. (**C**) Densitometry of cell counts. Data are expressed as the mean ± SEM (*n* = 3). Significant differences between the treatment and control groups are indicated as * (*p* < 0.05) or ** (*p* < 0.01).

### 2.4. Dicitrinone B Affects the Cell Cycle and the Integrity of the Plasma Membrane in A375 Cells

To further confirm dicitrinone B-induced apoptosis, cell cycle analysis and Annexin-V/PI double staining were performed. When A375 cells were treated with 5 μM and 10 μM of dicitrinone B, the percentage of G_2_/M cells increased from 3.56% in the control to 7.32% and 28.93%, respectively (Figure 4A). When the concentration of dicitrinone B reached 20 μM, the proportion of sub-G_0_/G_1_ cells, an important hallmark of apoptotic cells, significantly increased to greater than 50% (Figure 4A). These results suggested that dicitrinone B might inhibit A375 cell growth by blocking cells in the G_2_/M phase. Annexin-V/PI double staining results showed that early apoptosis rates of A375 cells changed from 15.80% to 30.70% to 20.30% and the late apoptotic cells’ increased from 23.10% to 43.60% to 60.80%, when treated with 5 μM, 10 μM and 20 μM of dicitrinone B (Figure 4B,C). However, when treated with 5-Fu, both the early and late cell apoptosis rates presented little change compared to the blank control group (Figure 4B,C). The above results demonstrate that dicitrinone B could effectively induce apoptosis by destroying the integrity of the plasma membrane, and the apoptosis rates presented in a dose-dependent manner.

### 2.5. Dicitrinone B Triggers Apoptosis through ROS Generation

**Figure 5:**
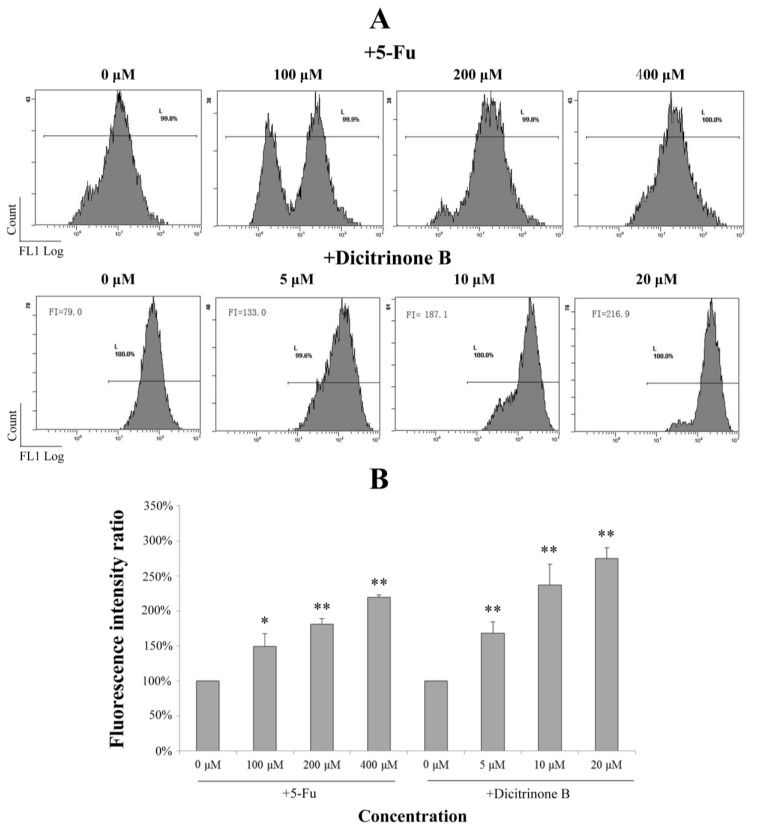
Dicitrinone B induces the accumulation of reactive oxygen species (ROS). (**A**) After dicitrinone B (five, 10 and 20 μM) treatment for 24 h, the level of intracellular ROS using DCF fluorescence was detected by flow cytometry, with 5-Fu (100, 200 and 400 μM) as a positive control. (**B**) Quantification of the results shown in (A). Values are expressed as the mean ± SD of three independent measurements. Significant differences between the treatment and control groups are indicated as * (*p* < 0.05) or ** (*p* < 0.01).

It has been reported that ROS accumulation could lead to mitochondrial dysfunction via depolarizing the mitochondrial membrane potential [15,16,17,18]. We next investigated whether dicitrinone B-induced apoptosis was triggered by ROS accumulation. DCFH-DA is a cell membrane permeable compound and is converted into the cell membrane impermeable nonfluorescent compound, DCFH, by intracellular esterases. Oxidation of DCFH by ROS produces a highly fluorescent DCF. The fluorescence intensity of DCF inside the cells is proportional to the amount of peroxide produced. Therefore, by using ROS-sensitive fluorescence dye DCFH-DA, we found that treatment of A375 cells with dicitrinone B and 5-Fu led to increased ROS accumulation in a dose-dependent manner (Figure 5). An average increase of 2–3 fold in ROS was observed under dicitrinone B treatment, while a comparable level was observed under twenty times concentration of the 5-Fu treatment. The data suggested that ROS may take part in the apoptosis of A375 cells induced by dicitrinone B.

**Figure 6:**
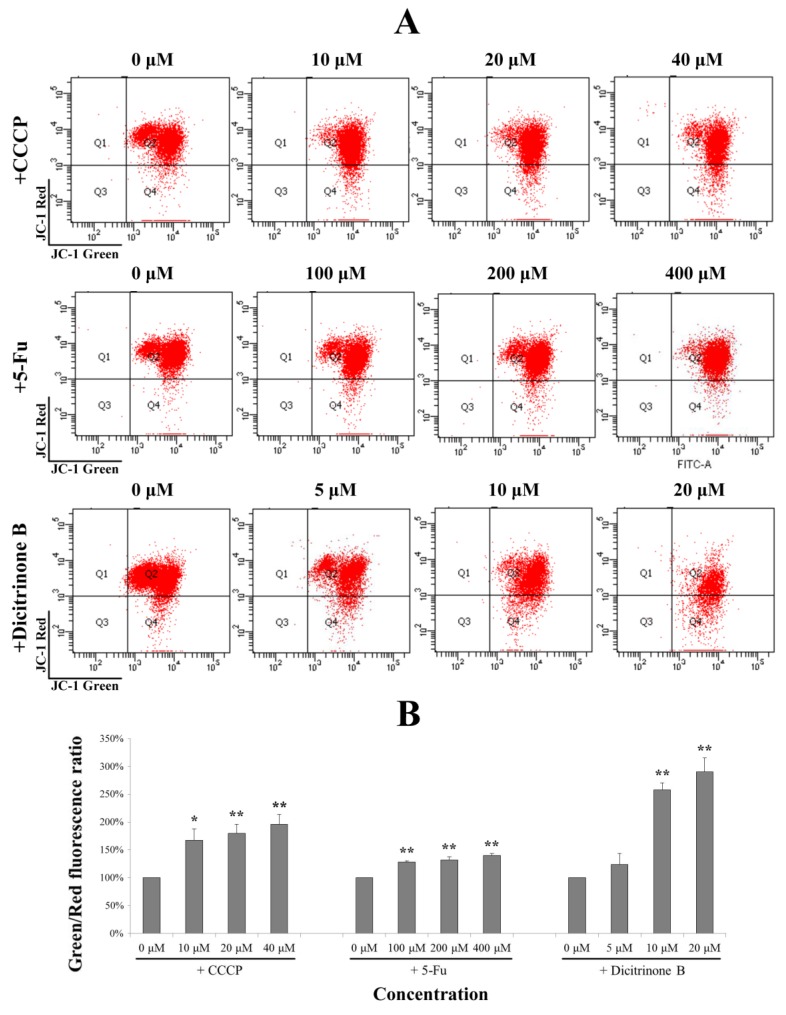
Dicitrinone B induces the loss of the mitochondrial membrane potential (MMP). (**A**) After dicitrinone B (zero, five, 10 and 20 μM) and 5-Fu (zero, 100, 200 and 400 μM) treatment for 24 h, cells were stained with JC-1 and then analyzed by flow cytometry. The ratio of green-to-red JC-1 fluorescence was used to quantitatively describe the decline of MMP, with CCCP (zero, 10, 20 and 40 μM) as a positive control. (**B**) Quantification of the results shown in (A). Values are expressed as the mean ± SD of three independent measurements. Significant differences between the treatment and control groups are indicated as * (*p* < 0.05) or ** (*p* < 0.01).

### 2.6. ROS Accumulation Caused by Dicitrinone B Treatment Leads to Mitochondria Damage

Once generated, ROS could cause mitochondrial membrane permeabilization [19]. Therefore, we examined the depolarization of the mitochondrial membrane potential using the fluorescent cationic dye, JC-1. In non-apoptotic cells, the negative charge established by the intact mitochondrial membrane potential allows the JC-1 to accumulate in the mitochondria and forms “J-aggregates” that become fluorescent red. By contrast, in apoptotic cells, the mitochondrial membrane potential collapses, and JC-1 remains in the cytoplasm in a monomeric form that exhibits green fluorescence [18]. The ratio of green-to-red JC-1 fluorescence was used to quantitatively describe the MMP. In order to evaluate the degree of dicitrinone B-reduced MMP, an MMP-disrupting agent, CCCP, was chosen as the positive control. As shown in Figure 6A, after being treated with gradient concentrations of dicitrinone B for 24 h, the ratio of green to red of JC-1 fluorescence was increased in a dose-dependent manner. Although there was no significant difference between the 5 μM of dicitrinone B treatment group and the control group, there was an average increase of 2–3 fold in the green-to-red JC-1 fluorescence ratio when exposed to 10 μM and 20 μM of dicitrinone B, and a higher level was presented compared to the 5-Fu and CCCP treatments (Figure 6B), indicating that dicitrinone B treatment leads to the loss of MMP.

### 2.7. Dicitrinone B Activates Caspase Pathway under the Regulation of Bcl-2 Family Proteins

**Figure 7:**
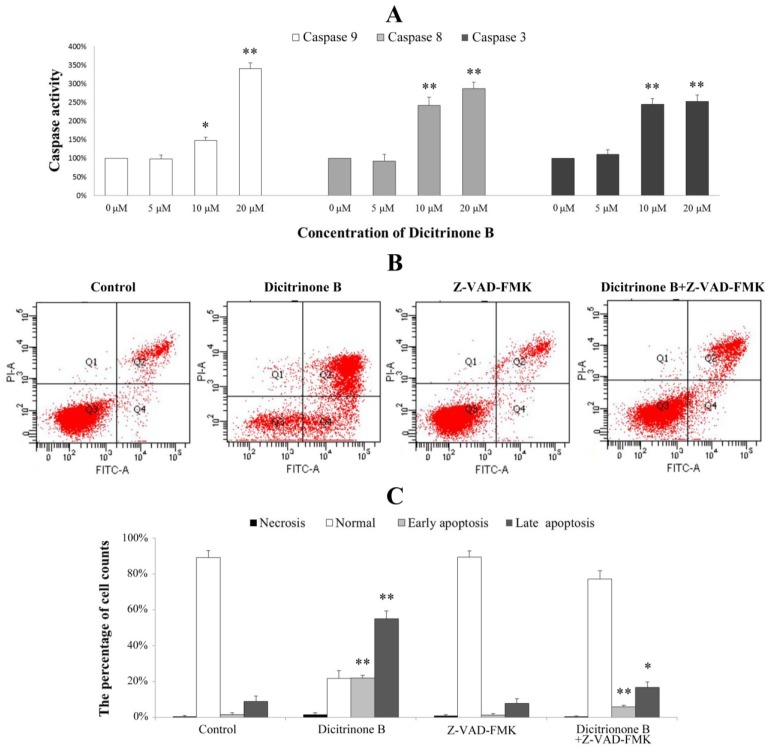
Analysis of the apoptosis-associated caspase activities in A375 cells. (**A**) A375 cells were treated with dicitrinone B (zero, five, 10 and 20 μM) for 24 h. The activities of caspase-9, -8 and -3 were determined by colorimetric assays kits, respectively. (**B**) A375 cells were incubated with 20 μM of dicitrinone B in the presence or absence of 20 μM of the caspase inhibitor, Z-VAD-FMK and then analyzed with Annexin-V/PI staining by flow cytometry. (**C**) Densitometry of cell counts. Data are expressed as the mean ± SD (*n* = 3). Significant differences between the treatment and control groups are indicated as * (*p* < 0.05) or ** (*p* < 0.01).

The caspase family is at the heart of apoptotic machinery, where these enzymes play key roles in the execution of apoptosis [20]. To identify whether caspases were involved in the mechanism, we measured the catalytic activity of caspase-9, caspase-8 and caspase-3 by colorimetric assays. The results demonstrated a gradual increase of cleaved caspase-9, cleaved caspase-8 and cleaved caspase-3 proteins in a dose-dependent manner, indicating that dicitrinone B could significantly activate caspase-9, caspase-8 and caspase-3 (Figure 7A). To further demonstrate the involvement of caspase activation in the apoptotic effect, we analyzed whether the pan-caspase inhibitor, Z-VAD-FMK, could prevent dicitrinone B-induced apoptosis. When A375 cells were incubated with 20 μM of dicitrinone B in the presence of 20 μM of the caspase inhibitor, Z-VAD-FMK, we observed that the apoptotic response was decreased significantly (Figure 7B,C). The proportion of late apoptotic cells decreased from 55.62% to 16.75%. This observation allowed us to conclude that there was involvement of the caspase-dependent pathways in the dicitrinone B-induced apoptotic death of A375 cells. 

**Figure 8:**
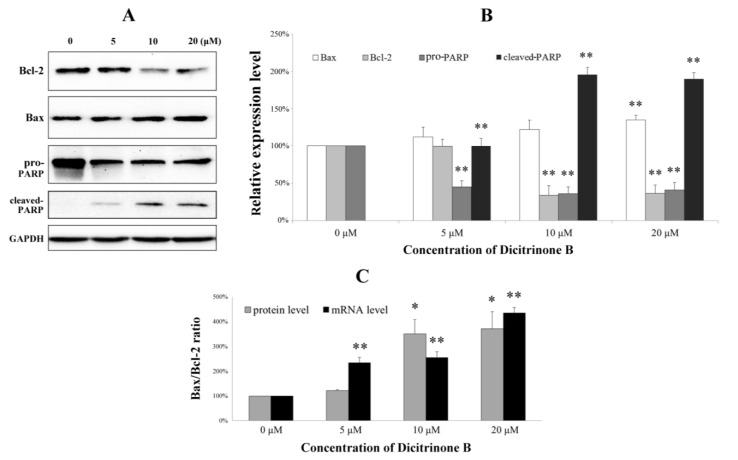
Real-time quantitative PCR and western blot analysis of the apoptosis-associated proteins in A375 cells. (**A**) After cells were treated with a different concentration of dicitrinone B for 24 h, the expressions of Bcl-2, Bax, pro-PARP and cleaved-PARP were determined by western blot, with GAPDH as a loading control. (**B**) Quantitative Bcl-2, Bax, pro-PARP and cleaved-PARP expressions after normalization to GAPDH. (**C**) The ratio of Bax/Bcl-2 at the protein and mRNA level in A375 cells treated with dicitrinone B (zero, five, 10 and 20 μM) for 24 h. Data are expressed as the mean ± SEM (*n* = 3). Significant differences between the treatment and control groups are indicated as * (*p* < 0.05) or ** (*p* < 0.01).

Since the activated caspase-3 could cleave PARP, which is an early and specific indicator of apoptosis [21], we next investigated pro-PARP and cleaved-PARP expression by western blot (Figure 8A). We found that pro-PARP decreased and cleaved-PARP increased both in a dose-dependent manner, indicating that PARP was cleaved during the process of apoptosis induced by dicitrinone B. Although the caspase proteolytic cascade is a central point in the apoptotic response, its activity is tightly regulated by a variety of factors. Bcl-2 family proteins, including antiapoptotic members (such as Bcl-2) and proapoptotic members (such as Bax) have been proven to be one of the most important factors and play a pivotal role [21,22]. To further analyze the possible mechanism underlying dicitrinone B-induced apoptosis, we tested the expression of Bcl-2 and Bax by western blot. The results showed that the Bax level increased while the Bcl-2 level decreased in a dose-dependent manner (Figure 8A,B). The ratio of Bax/Bcl-2, which is a key factor regulating apoptosis, was further calculated and proven to increase in a dose-dependent manner. Furthermore, the mRNA levels of Bax and Bcl-2 were determined by real-time quantitative PCR. After 24 h of dicitrinone B treatment, the ratio of Bax/Bcl-2 mRNA was increased with the increased concentration (Figure 8C), which was consistent with the previous data of the western blot analysis. To summarize, dicitrinone B induces the apoptosis of A375 cells by activating the caspase-cascade response and regulating the expression of Bax and Bcl-2.

### 2.8. Global Discussion

It was reported that the specific situations that microorganisms live in might activate some silent genes and induce some unique biosynthetic pathways [23]. Marine fungi are an important resource for finding chemically and biologically diverse compounds, due to their special living environment. In recent years, interest in the bioactive ingredients of marine fungi has been growing rapidly, as most of the fungal-derived compounds are capable of inhibiting the growth and proliferation of cancer cells. In this study, we obtained dicitrinone B, a novel carbon-bridged citrinin dimer, isolated and purified using many purification methods, from the marine-derived fungus, *Penicillium citrinum*, and tested for its cytotoxicity effect on multiple tumor cells. The results showed that dicitrinone B effectively inhibited the cell growth of multiple tumor cells, especially A375 cells. Most interestingly, it seems to have more potent anticancer efficacy, inhibiting cell proliferation and tumor growth, as compared to large doses of the first-line chemotherapy drug, 5-Fu, in A375 cells. Further study indicated that dicitrinone B induced apoptosis, at least in part, mediated by two apoptosis pathways. The first one was through the ROS-related intrinsic mitochondrial pathway, and the second one was through the extrinsic apoptotic pathway. This is the first report that clearly characterizes the antitumor properties of dicitrinone B and identifies its mechanism in a tumor model.

In our study, we found that dicitrinone B pretreatment observably induced rapid ROS generation in a dose-dependent manner. Increased oxidative stress could cause the opening of the mitochondrial permeability transition pore (MPTP mitochondrial) and eventually leads to mitochondrial membrane permeabilization [24]. Mitochondria are vital intracellular organelles, whose functions encompass, but are not limited to, ATP production, apoptosis regulation and biosynthesis of several metabolites. They are also the main cellular source and immediate target of ROS [25]. We found the attenuation of MMP after dicitrinone B treatment and speculated that it was the result of the excessive accumulation of ROS. Once the mitochondria are destabilized, the apoptogenic factors, such as cytochrome c, would be released from the outer mitochondria membrane space into the cytosol [26]. It has been reported that cytochrome c release induces caspase activation, which, in turn, may promote either cell death or vital processes, like differentiation and proliferation. These opposite outcomes may derive from different subsets of substrates, which are cleaved by the caspases in different situations [25]. Although we have not detected the release of cytochrome c in our study yet, we would do more in-depth research in the future.

We further detected the activation of caspase-9, caspase-8 and caspase-3, which play key roles in the execution of apoptosis and the cleavage of PARP, indicating the subsequent induction of both the extrinsic and intrinsic apoptosis pathways. Regardless of the extrinsic or intrinsic apoptotic pathway, caspase-3 is the core protein involved in cellular apoptosis [27]. The extrinsic pathway is regulated by a death-inducing signaling complex, which is made of a Fas-associated death domain and procaspase-8. The death-inducing signaling complex activates caspase-8 and the downstream caspases [15]. It has been reported that ROS accumulation could activate ASK1 to participate in the process of the extrinsic apoptotic pathway [28]. We detected the activation of caspase-8, suggesting that extrinsic apoptosis pathways may also contribute to dicitrinone B-induced apoptosis. However, the precise mechanism of this pathway remains to be determined.

Members of the Bcl-2 family are major regulators of cell death or cell survival [29]. Bcl-2 family proteins include both pro-apoptotic members (e.g., Bax, Bid, Bad and Bim) and anti-apoptotic members (e.g., Bcl-2, Bcl-XL, Mcl-1 and Bcl-w). The balance of pro-apoptotic and anti-apoptotic Bcl-2 family proteins decides the fate of the cell [30]. Our data clearly showed that dicitrinone B treatment of A375 cells resulted in a dose-dependent increase in the level of Bax with a concomitant decrease in the Bcl-2 level and, finally, caused an increase of the Bax/Bcl-2 ratio, demonstrating that Bcl-2 family protein regulation would be involved in dicitrinone B-induced apoptosis. It has been reported that Bcl-2 family proteins could regulate the caspase cascade [31]. Some studies highlighted the importance of the Bc1-2 family in protecting mitochondria against the loss of function during apoptosis [32], while other opinions on the Bc1-2 family focused on its inhibiting of the release of the apoptosis-associated factor, cytochrome c, from the mitochondria [33] or regulating ion flux [34]. Our data suggested that Bcl-2 and Bax participated in dicitrinone B-induced apoptosis, but the details of the mechanisms need further study. 

## 3. Experimental Section

### 3.1. Preparation of Dicitrinone B

#### 3.1.1. Fungal Material

The fungus, *Penicillium citrinum*, was isolated from marine sediments collected from Langqi Island, Fujian, China. It was identified according to its morphological characteristics and ITS by Beijing Sunbiotech Co. Ltd, (Beijing, China) and preserved in our laboratory at −80 °C. The producing strain was prepared on Martin medium and stored at 4 °C.

#### 3.1.2. Fermentation and Extraction

The fungus was cultured under static conditions at 28 °C for 30 days in 1000-mL conical flasks containing the liquid medium (400 mL/flask) composed of glucose (20 g/L), peptone (5 g/L), malt extract (3 g/L), yeast extract (3 g/L) and seawater after adjusting its pH to 7.0. The fermented whole broth (100 L) was filtered through cheese cloth to separate the supernatant from mycelia. The former was extracted two times with EtOAc to yield an EtOAc solution that was concentrated under reduced pressure to give a crude extract (58.0 g).

#### 3.1.3. Purification of Dicitrinone B

The crude extract (58.0 g) was separated into 7 fractions on a Si gel column using a step gradient elution of petroleum ether, CH_2_Cl_2_ and MeOH. Fraction 3 (7.5 g) eluted with petroleum ether/CH_2_Cl_2_ (1:3) was further purified on a Si gel column using a step gradient elution. Subfraction 3–5 (1.3 g) eluted with petroleum ether/EtOAc (1:1) was purified on a Sephadex LH-20 (CH_2_Cl_2_/MeOH, 1:1) and a reversed-phase column (MeOH/H_2_O, 2:1) to give dicitrinone B (310 mg). 

### 3.2. Culture of Cell Lines

Twenty tumor cell lines derived from eight different types of tumors were used, including: liver cancer cell lines HuH-7, HepG2 and PLC/PRF/5; gastric cancer cell lines BGC-823, SGC-7901 and HGC-27; lung cancer cell lines SPC-A1, 95-D and SK-MES-1; malignant melanoma cell lines A375; breast cancer cell lines MCF-7 and SK-BR-3; esophagus cancer cell lines EC-9706 and KYSE450; colon cancer cell lines LoVo, SW480 and SW620; and lymphoma cell lines U-937, Raji and Jeko-1. All these cell lines were obtained from Shanghai Cell Resource Center. HuH-7 and A375 were cultured in DMEM (Hyclone) medium; HepG2, HGC-27 and SK-MES-1 were cultured in EMEM (Hyclone) medium; and the rest of the cell lines were cultured in RPMI 1640 (Takara) medium. The medium was supplemented with 10% fetal bovine serum (Hyclone), and the cells were cultured at 37 °C in a 5% CO_2_ atmosphere.

### 3.3. WST-1 Cell Proliferation Assay

Cytotoxic activity was evaluated by the WST-1 cell proliferation assay kit (Roche, Mannheim, Germany). Briefly, cells were cultured in a 96-well plate and treated with gradient concentrations of dicitrinone B or the positive control. After 48 h, one-tenth of the volume of the WST-1 solution was added. After cells were incubated at 37 °C for 4 h, the absorbance at 450 nm was measured. 

### 3.4. Morphological Analysis

#### 3.4.1. Hoechst 33258 Staining

Hoechst labeling of cells was used to detect apoptotic nuclei by the evaluation of nuclear morphology, which was performed as previously described [8]. Briefly, A375 cells were incubated with gradient concentrations of dicitrinone B for 24 h and then fixed in a solution of methanol and acetic acid with 3:1 for 15 min at room temperature and stained with Hoechst 33258 at a final concentration of 10 μg/mL for 15 min. After being washed with PBS twice, the cells were observed under an Olympus fluorescence microscope (Olympus, Tokyo, Japan). 

#### 3.4.2. AO/EB Staining

AO/EB double staining was used to detect the apoptosis of A375, as previously described [9]. Briefly, after the desired treatments, cells were harvested and stained with AO/EB solution containing 100 µg/mL AO and 100 µg/mL EB for 15 min, then washed twice with PBS and observed under an Olympus fluorescence microscope.

### 3.5. Flow Cytometry

#### 3.5.1. Cell Cycle Analysis

Briefly, cells were incubated with gradient concentrations of dicitrinone B for 24 h and then collected, permeabilized and fixed with cold 70% ethanol at 4 °C overnight. After being washed with cold PBS, cells were resuspended in 1.0 mL PI solution containing 100 µg/mL of DNase-free RNase A and 50 µg/mL of propidium iodide and then measured by flow cytometry (BD Biosciences, San Jose, CA, USA) to detect apoptosis. A minimum of 10,000 events were analyzed in each experiment.

#### 3.5.2. Annexin V/PI Staining

Apoptosis assays were performed as previously described [10] by using the Annexin V FLUOS staining Kit (Roche, Mannheim, Germany). Briefly, after the desired treatments, cells were collected and washed twice with binding buffer and then resuspended at a concentration of 1 × 10^6^ cells/mL in binding buffer. Five hundred microliters of the cell suspension were mixed with 5 μL of Annexin V-EGFP and 5 μL of propidium iodide. After being incubated at room temperature for 15 min, apoptotic cells were determined using a FACScan (BD Biosciences, San Jose, CA, USA).

The pan-caspase inhibitor, Z-VAD-FMK (Beyotime, Haimen, China), was added to cells at the final concentration of 20 μM before dicitrinone B treatment.

#### 3.5.3. ROS Assay

Intracellular ROS production was performed as previously described [10] by using the Reactive Oxygen Species Assay Kit (Beyotime, Haimen, China). Briefly, after the desired treatments, cells were harvested, washed twice with PBS and then incubated in serum-free DMEM containing the reagent, DCFH-DA (10 μM), for 20 min at 37 °C. Then, cells were washed with serum-free DMEM and immediately submitted to flow cytometric analysis using a FACScan flow cytometer (BD Biosciences, San Jose, CA, USA). A minimum of 10,000 events were analyzed in each experiment.

#### 3.5.4. MMP Assay

The MMP was performed as previously described [10] by the MMP probe JC-1 dye, which emits red fluorescence under high MMP and green fluorescence under low MMP conditions. The MMP assay was carried out according to the mitochondrial membrane potential assay kit with JC-1 (Beyotime, Haimen, China). Briefly, after the desired treatments, cells were harvested, washed twice with PBS and then resuspended in staining buffer containing JC-1 (1×). After being incubated at 37 °C for 20 min, cells were washed with staining buffer (1×) twice and submitted to flow cytometric analysis using a FACScan flow cytometer. The MMP-disrupting agent, CCCP, was added to cells at the concentration of 10, 20 and 40 μM as a positive control (Beyotime, Haimen, China). The green and red fluorescence ratio measured the proportion of mitochondrial depolarization. A minimum of 10,000 events were analyzed in each experiment. 

### 3.6. Western Blot

Total proteins were extracted by RIPA buffer containing a cocktail of protein inhibitors (Roche, Mannheim, Germany). The protein concentration was determined by the Bicinchoninic Acid assay (Beyotime, Haimen, China). Equal amounts of protein were separated by 12% SDS-PAGE and then transferred to the PVDF membrane (Bio-Rad, Hercules, CA, USA). Proteins were detected using primary antibodies against Bax (1:1000, Cell Signaling Technology, Boston, USA), Bcl-2 (1:1000, cell signaling technology, Boston, MA, USA), pro-PARP (1:1000, Sangon, Shanghai, China), cleaved-PARP (1:400, sc-23461-R, Santa Cruz Biotechnology, Santa Cruz, CA, USA) and GAPDH (1:1000, Sangon, Shanghai, China). The secondary antibodies were peroxidase-conjugated anti-rabbit IgG or anti-mouse IgG (1:10,000, Thermo Scientific Pierce, Rockford, IL, USA). Signals were detected by an ECL system (Beyotime, Haimen, China) using a FluorChem E digital darkroom system (Protein Simple, Santa Clara, CA, USA). 

### 3.7. Caspase Activity Assay

The activities of caspase-9, -8 and -3 were determined using colorimetric assays kits (Beyotime, Haimen, China) according the manufacturer’s protocol. Briefly, after the desired treatments, cells were harvested and lysed in lysis buffer. The cell lysate was used to estimate the caspase-9, -8 and -3 activities. Assays were performed on a 96-well plate by incubating 10 μL of the protein of the cell lysate per sample in 80 μL of reaction buffer, which contained caspase-9, -8 or -3 substrates. After incubation at 37 °C for 4 h, the formation of *p*-nitro-anilide was measured at 405 nm.

### 3.8. Real-Time Quantitative RT-PCR

After the desired treatments, total RNA was extracted from the cultured cells using the acid guanidinium thiocyanate-phenol-chloroform method (TRIzol, Invitrogen, Carlsbad, CA, USA), and cDNAs were synthesized using the PrimeScriptTM RT reagent kit (Takara, Dalian, China). Quantitative PCR was performed using the Bio-Rad CFX96 Real-Time PCR Detection System with SYBR^®^Premix Ex TaqTM (Tli RNaseH Plus) (Takara, Dalian, China), and the primers are listed in Table 1.

**Table 1:** Primers for real-time quantitative RT-PCR.

Gene Names	Sequences	
Bax	forward	5′-CCTTTTCTACTTTGCCAGCAAAC-3′
	reverse	5′-GAGGCCGTCCCAACCAC-3′
Bcl-2	forward	5′-CGACTTTGCAGAGATGTCCA-3′
	reverse	5′-ATGCCGGTTCAGGTACTCAG-3′
GAPDH	forward	5′-GAAGGTGAAGGTCGGAGTC-3′
	reverse	5′-GAAGATGGTGATGGGATTTC-3′

The mRNA expression levels were calculated using the 2^−ΔΔCT^ method and expressed in relative quantification units. A control without cDNA was run in parallel with each assay. Each reaction was amplified in triplicate, and relative mRNA levels were normalized to those of GAPDH.

### 3.9. Statistical Analysis

The data were presented as the means ± SD. Statistical analyses were performed using the Student’s *t*-test and one way analysis of variance. All computations were done by employing statistical software (SAS, version 8.0, SAS Institute Inc., Cary, NC, USA). Values of *p* < 0.05 and *p* < 0.01 were accepted as an indication of statistical significance.

## 4. Conclusions

**Figure 9:**
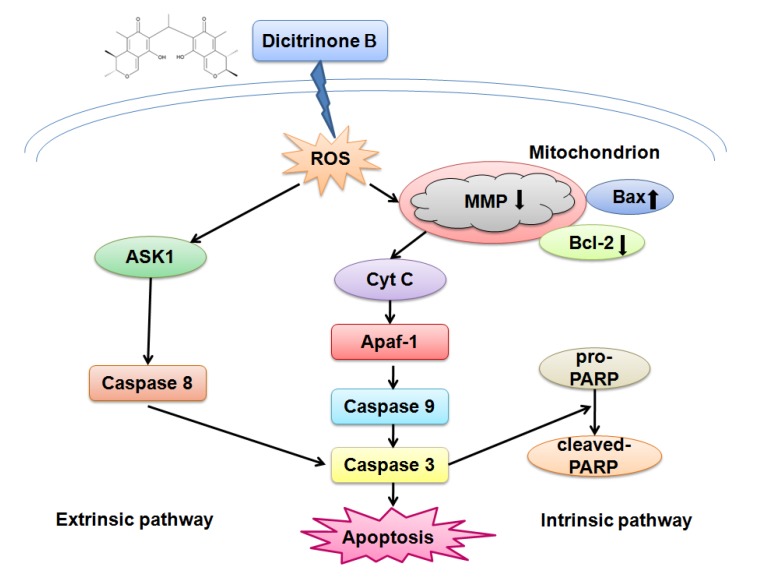
The proposed signaling pathways for dicitrinone B-induced apoptosis in A375 cells. The effect of apoptosis is most likely achieved through rapid ROS generation, which, at least in part, leads to mitochondrial membrane permeabilization and triggers the intrinsic apoptosis pathway, as well as the extrinsic apoptosis pathway.

In this work, we described how dicitrinone B significantly inhibited the growth of multiple tumor cells and induced A375 cell apoptosis *in vitro*. The apoptotic mechanism was related to the ROS-mediated intrinsic and extrinsic apoptotic pathways, including the activation of caspase-9, caspase-8 and caspase-3, which were mediated by Bcl-2 family proteins. A proposed mechanism of the activity displayed by dicitrinone B can be found in Figure 9. Further work should be performed using *in vivo* models to better understand the role of body toxicology.

## Supplementary Files

Supplementary File 1 Supplementary Information (PDF, 684 KB)

## Abbreviations

5-Fu: 5-fluorouracil
AO/EB: acridine orange/ethidium bromide
ASK1: apoptosis signal-regulating kinase 1
CCCP: carbonyl cyanide 3-chlorophenylhydrazone
DCF: 2′,7′-dichlorofluorescein
DCFH-DA: dichlorodihydrofluorescein diacetate
DMEM: Dulbecco’s Modified Eagle’s Medium
EGFP: enhanced green fluorescent protein
EMEM: Eagle’s minimal essential medium
EtOAc: ethyl acetate
G2/M: gap 2/mitosis phase
GAPDH: glyceraldehyde-3-phosphate dehydrogenase
HPLC: high performance liquid chromatography
HRESIMS: high-resolution electrospray ionization mass spectroscopy
ITS: internal transcribed spacer
JC-1: 5,5′,6,6′-tetrachloro-1,1′,3,3′-tetraethyl-imidacarbocyanine iodide
MMP: mitochondrial membrane potential
NMR: nuclear magnetic resonance
PARP: poly(ADP-ribose) polymerase
PBS: phosphate-buffered saline
PI: propidium iodide
PVDF: polyvinylidene fluoride
RIPA: radio-immunoprecipitation assay
ROS: reactive oxygen species
RPMI: Roswell Park Memorial Institute
RT: reverse transcription
WST-1: 2-(4-Iodophenyl)-3-(4-nitrophenyl)-5-(2,4-disulfophenyl)-2*H*-tetrazolium monosodium salt
