# Supplementary material for: The Marine Fungal Metabolite, Dicitrinone B, Induces A375 Cell Apoptosis through the ROS-Related Caspase Pathway
Source: Mar Drugs. 2014 Apr 2;12(4):1939–58. doi: 10.3390/md12041939 (PMC4012433; doi:10.3390/md12041939)
Supplement: Supplementary File 1 — Supplementary Information (PDF, 684 KB) [file marinedrugs-12-01939-s001.pdf]

Supplementary Information

Figure S1. The purity analysis of dicitrinone B by HPLC. (**A**) 80% MeCN/H_2O, 0.1% TFA, 343 nm. (**B**) 80% MeCN/H_2O, 0.1% TFA, 201 nm. (**C**) 80% MeCN/H_2O, 0.1% TFA, DAD.

A

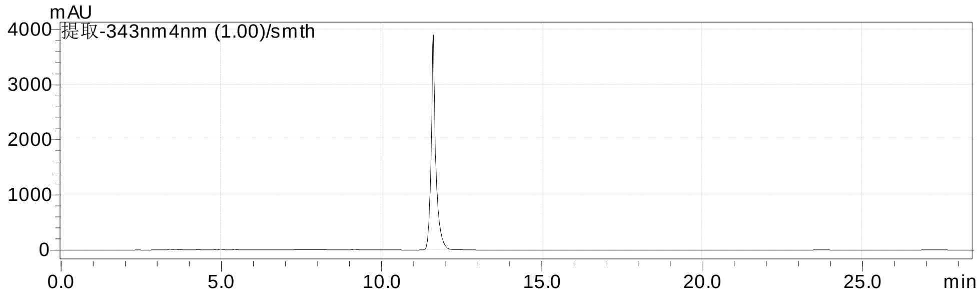

B

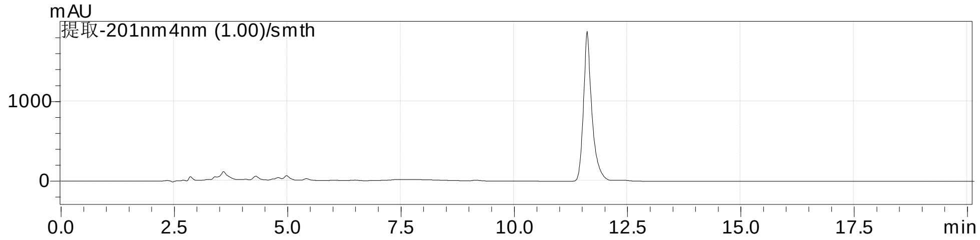

C

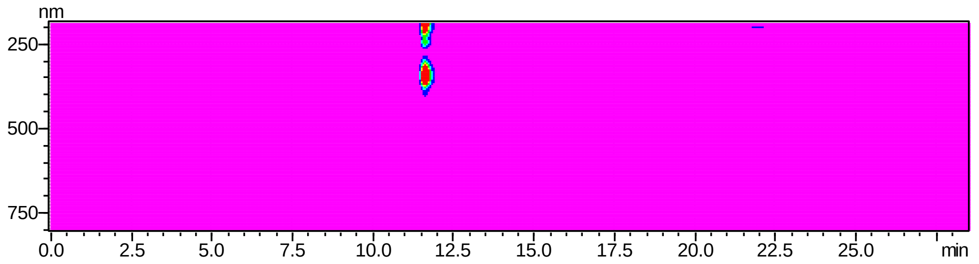

Figure S2. HRESIMS spectrum of dicitrinone B.

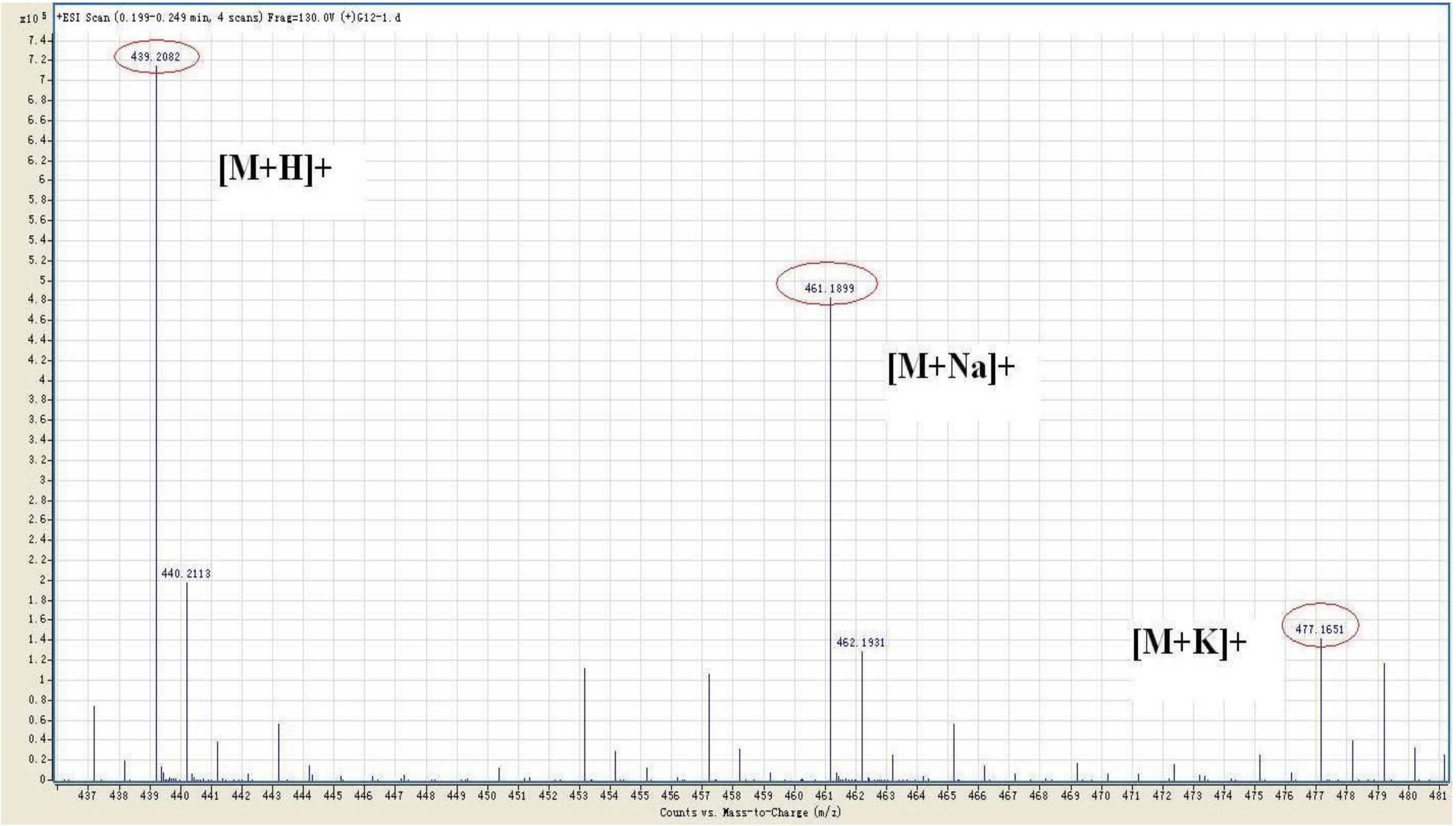

Figure S3. ^{1}H NMR (500 MHz, DMSO-d_6) spectrum of dicitrinone B.

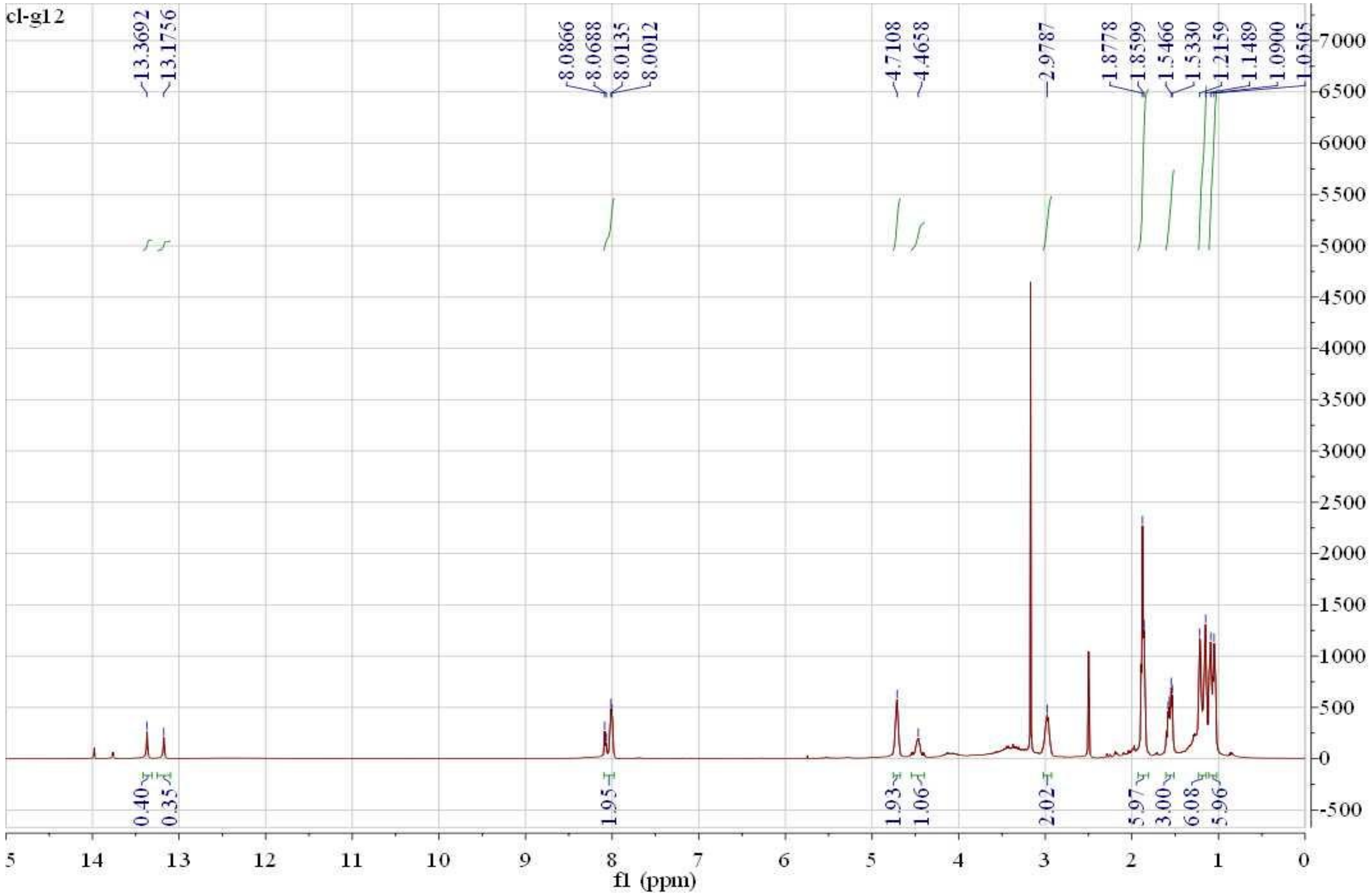

Figure S4. ^{13}C NMR (125 MHz, DMSO-d_6) spectrum of dicitrinone B.

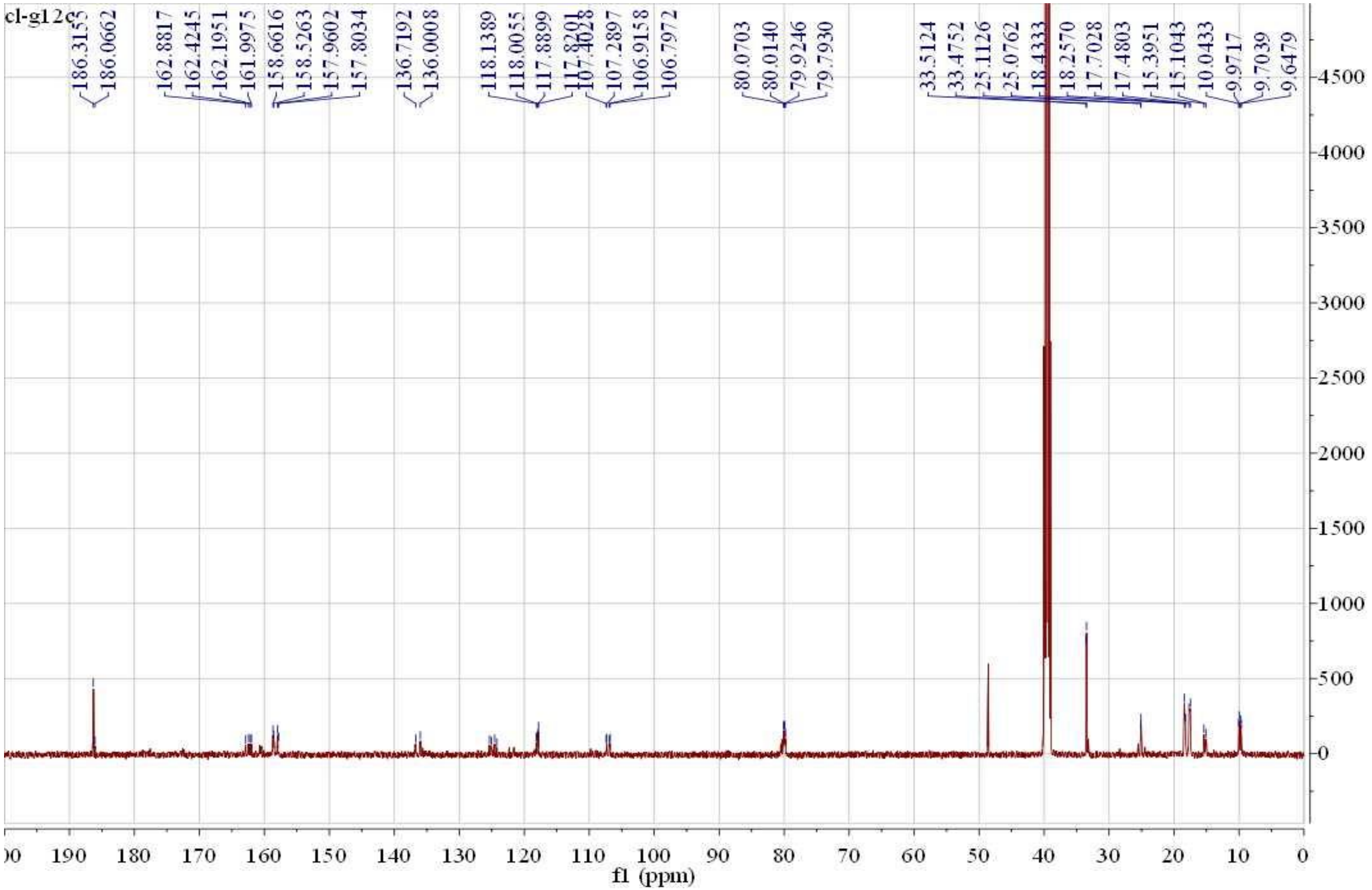

© 2014 by the authors; licensee MDPI, Basel, Switzerland. This article is an open access article distributed under the terms and conditions of the Creative Commons Attribution license (http://creativecommons.org/licenses/by/3.0/).
